# Genome-wide identification of the soybean cytokinin oxidase/dehydrogenase gene family and its diverse roles in response to multiple abiotic stress

**DOI:** 10.3389/fpls.2023.1163219

**Published:** 2023-04-17

**Authors:** Yanli Du, Zhaoning Zhang, Yanhua Gu, Weijia Li, Weiyu Wang, Xiankai Yuan, Yuxian Zhang, Ming Yuan, Jidao Du, Qiang Zhao

**Affiliations:** ^1^ Agricultural College, Heilongjiang Bayi Agricultural University, Daqing, Heilongjiang, China; ^2^ National Cereals Technology Engineering Research Center, Daqing, Heilongjiang, China; ^3^ Heilongjiang Bayi Agricultural University, Key Laboratory of Ministry of Agriculture and Rural Affairs of Soybean Mechanized Production, Daqing, Heilongjiang, China; ^4^ Qiqihar Branch of Heilongjiang Academy of Agricultural Sciences, Qiqihar, Heilongjiang, China; ^5^ Research Center of Saline and Alkali Land Improvement Engineering Technology in Heilongjiang Province, Daqing, Heilongjiang, China

**Keywords:** abiotic stress, CKX gene family, soybean, expression analysis, growth and development

## Abstract

Cytokinin oxidase/dehydrogenase (CKX) irreversibly degrades cytokinin, regulates growth and development, and helps plants to respond to environmental stress. Although the *CKX* gene has been well characterized in various plants, its role in soybean remains elusive. Therefore, in this study, the evolutionary relationship, chromosomal location, gene structure, motifs, *cis*-regulatory elements, collinearity, and gene expression patterns of *GmCKXs* were analyzed using RNA-seq, quantitative real-time PCR (qRT-PCR), and bioinformatics. We identified 18 *GmCKX* genes from the soybean genome and grouped them into five clades, each comprising members with similar gene structures and motifs. *Cis*-acting elements involved in hormones, resistance, and physiological metabolism were detected in the promoter regions of *GmCKXs*. Synteny analysis indicated that segmental duplication events contributed to the expansion of the soybean CKX family. The expression profiling of the *GmCKXs* genes using qRT-PCR showed tissue-specific expression patterns. The RNA-seq analysis also indicated that *GmCKXs* play an important role in response to salt and drought stresses at the seedling stage. The responses of the genes to salt, drought, synthetic cytokinin 6-benzyl aminopurine (6-BA), and the auxin indole-3-acetic acid (IAA) at the germination stage were further evaluated by qRT-PCR. Specifically, the *GmCKX14* gene was downregulated in the roots and the radicles at the germination stage. The hormones 6-BA and IAA repressed the expression levels of *GmCKX1*, *GmCKX6*, and *GmCKX9* genes but upregulated the expression levels of *GmCKX10* and *GmCKX18* genes. The three abiotic stresses also decreased the zeatin content in soybean radicle but enhanced the activity of the CKX enzymes. Conversely, the 6-BA and IAA treatments enhanced the CKX enzymes’ activity but reduced the zeatin content in the radicles. This study, therefore, provides a reference for the functional analysis of *GmCKXs* in soybean in response to abiotic stresses.

## 1 Introduction

Soybean (*Glycine max* L.) is an important food and oil crop worldwide, with its seed oil accounting for approximately 30% of the global vegetable oil consumption (Zhan et al., 2020). Soybean seeds contain various substances beneficial to human health, which have been proven important in preventing and treating cancer, atherosclerosis, osteoporosis, and coronary heart disease (Malenčić et al., 2012; Zhang et al., 2014). Soybeans also have a variety of industrial uses and are considered a potential crop for biodiesel production (Woyann et al., 2019). In the US, 30% of printed matter uses soybean ink, and many city buses have switched to an environmentally friendly blend of soybean oil and diesel (Frederick et al., 2001). Brazil, the world’s top soybean producer, reportedly produced 124.5 million tons during 2019–2020 (USDA, 2020). Furthermore, soybean is one of the most common crops in arid and semi-arid areas where its growth and yield are easily affected by various abiotic stresses (Silvente et al., 2012; Gavili et al., 2019). Therefore, there is a need to explore the molecular mechanism involved in the soybean response to abiotic stress.

Cytokinin (CTK) is an important plant hormone that regulates many plant growth and development processes. CTKs naturally occurring in plants are derivatives of adenine and contain an isoprene-derived side chain or an aromatic side chain at the N^6^ end, called isoprenoid CTKs and aromatic CTKs (**Figure S1**), respectively (Sakakibara, 2006). In plants, CTKs are distributed mainly in the dividing cells of stem and root tips, immature and germinating seeds, and growing fruits, promoting cell division and regulating differentiation. In tissue culture, the high ratio of CTKs to auxin benefits shoot differentiation, while a low ratio promotes root differentiation. The phytohormone also delays protein and chlorophyll degradation and plays a role in response to biological and abiotic stresses (Hwang et al., 2012; Jameson & Song, 2016; Wybouw & De Rybel, 2019).

Cytokinin oxidation/dehydrogenase (CKX) enzymes are encoded by a family of CKX genes that can specifically degrade unsaturated side chains at the N^6^ position in CTKs and catalyze their irreversible degradation (Brownlee et al., 1975; McGaw & Horgan, 1983; Schmülling et al., 2003). Multiple gene families encoding CKX proteins (Werner et al., 2006) have been identified, and their evolution has been extensively studied in *Arabidopsis thaliana* (Werner et al., 2003), *Oryza sativa* (Ashikari et al., 2005; Rong et al., 2022), *Nicotiana tabacum* (Rong et al., 2022), *Zea mays* (Zalabák et al., 2014), *Triticum aestivum* (Mameaux et al., 2012), *Brassica rapa* (Mameaux et al., 2012), *Brassica napus* (Liu et al., 2018), *Brassica oleracea* (Zhu et al., 2022), and *Vitis vinifera* (Yu et al., 2021). The *CKX* genes also have several other functions in plants. For example, compared with the wild type, *atckx3/ckx5* double mutant showed increased stem apex meristem and silique number of *Arabidopsis* (Bartrina et al., 2011), while the inhibition of the expression of *OsCKX2* promotes rice growth by increasing its tiller number and yield (Gao et al., 2014; Yeh et al., 2015). The *OsCKX11* gene also regulates leaf senescence and grain number and coordinates the source–sink relationship in rice (Zhang et al., 2021). The *CKX* genes are also involved in plant responses to various biological and abiotic stresses. For example, suppressing the expression of the *CKXs* gene can significantly enhance *Arabidopsis* resistance against *Verticillium* wilt and fungal infection (Reusche et al., 2013). *Bol020547*, *Bol028392*, and *Bol045724* are important in determining cabbage (*B. oleracea* var. *capitata*) tolerance to *Plasmodiophora brassicae* (Zhu et al., 2022). In maize, most *CKX* genes were upregulated under salt stress (Vyroubalová et al., 2009); the overexpression of *CKXs* genes also enhanced *Arabidopsis* and tobacco tolerance to drought, salt, and abscisic acid stress (Nishiyama et al., 2011; Werner et al., 2011).

In this study, the *CKX* gene family in the whole soybean genome was identified and analyzed by bioinformatics techniques. The gene structure, chromosome distribution, *cis*-regulatory elements, gene replication, collinearity, and spatiotemporal expression patterns of the *GmCKX* genes were further analyzed. In addition, the key *GmCKX* genes that respond to salt, drought, salt combined with drought stress, 6-benzylaminopurine (6-BA), and indole-3-acetic acid (IAA) were screened. The results of this study lay the foundation for the study of *GmCKXs* gene function and provide important information for elucidating the evolutionary roles of *CKXs*.

## 2 Materials and methods

### 2.1 Identification and analysis of the *GmCKX* genes

The information of the reference genome and annotated proteins of soybean (*Glycine max* Wm82.a2.v1) was obtained from Ensembl Plants (http://plants.ensembl.org/index.html). The hidden Markov model (HMM) profile (http://hmmer.janelia.org/) and the Pfam database (http://pfam-legacy.xfam.org/) were used to screen candidate GmCKX proteins (PF01565 and PF09265). The CKXs protein sequence files were obtained from Ensembl Plants database (http://plants.ensembl.org/index). The InterPro (http://www.ebi.ac.uk/interpro/) (Finn et al., 2017) and SMART (http://smart.embl-heidelberg.de/) (Letunic et al., 2015; Han et al., 2019) software were used to further confirm the reliability of the CKX domain prediction. Then, the integrity of CKX domains was confirmed by Prosite (http://prosite.expast.org/) and WoLF PSORT (http://wolfpsort.hgc.jp/). All identified *GmCKX* genes were mapped according to their reference genome and named according to their locations on the chromosome using TBtools (Chen et al., 2020a). The CKX protein sequences derived from *Arabidopsis*, maize (*Z. mays* L.), and rice (*O. sativa* L.) were obtained from Ensembl Plants by searching CKX domains and used for phylogenetic analysis. A phylogenetic tree was constructed using the maximum likelihood method with 1,000 bootstrap replicates and the JTT+G model by MEGA X (version X-10.1.8, Mega Limited, Auckland, New Zealand).

The exon–intron structure of *GmCKX* genes was analyzed by the GSDS platform (http://gsds.cbi.pku.edu.cn/) (Guo, 2007). Gene-wise (Birney et al., 2004) was used to detect the correspondence between DNA and protein sequences. Then, the CKX domain coordinates in the protein sequence were converted to the coordinates in the nucleotide sequence using in-house perl script. The conserved motifs of CKX proteins were analyzed using MEME tool (http://meme.nbcr.net/meme/) (Bailey et al., 2009) with the following parameters: the motif length set at 10–50 amino acids and E value < 1e^−20^. The upstream regions (1,500 bp) of *GmCKX* genes were extracted and used as the gene promoter sequence. The *cis*-regulatory elements were analyzed by the PlantCare database (https://bioinformatics.psb.ugent.be/webtools/plantcare/html/). The Multiple Collinearity Scan toolkit (MCScanX) was used to analyze the synteny and collinearity of *GmCKX* genes (Wang et al., 2012). Subsequently, the collinearity of the duplicated genes was visualized by Circos software (version 0.69) (Krzywinski et al., 2009). The expression data of *GmCKXs* in different tissues came from the Phytozome database (https://phytozome-next.jgi.doe.gov) and Soybean ePF Browser database (http://bar.utoronto.ca/efpsoybean/cgi-bin/efpWeb.cgi), respectively. The heatmap was generated using TBtools (Chen et al., 2020a).

### 2.2 Plant materials and treatments

The soybean seeds (Heike68) were obtained from the National Coarse Cereals Engineering Research Center, Daqing, Heilongjiang, China. The surface-sterilized soybean seeds were placed on a petri dish measuring 9 cm in diameter and incubated in the dark at 28°C until germination, which was indicated by the emergence of radicles. After 5 days of germination under distilled water treatment (CK), samples of soybean cotyledons, radicles, and hypocotyls were harvested, frozen in liquid nitrogen for 5 min, and then stored at −80°C for tissue-specific expression analysis of *GmCKXs* using quantitative real-time PCR (qRT-PCR). After 4 days of germination, we selected seedlings with consistent growth to explore the response of their *GmCKXs* to different abiotic stress. The experimental treatments consisted of seedlings exposed to 150 mM NaCl (SS, simulated salt stress), 20% (W/V) PEG 6000 (D, simulated drought stress), 150 mM NaCl +20% (W/V) PEG 6000 (SS+D), 10 µM IAA, and 10 µM 6-BA. The seedlings were exposed to treatments as described previously (Liu et al., 2018), with those treated with distilled water (CK) alone as controls. We then incubated the treated seedlings at 28°C for 24 h in the dark, harvested radicle samples from the treated seedlings in liquid nitrogen, and then stored them at −80°C before further use.

Average-sized soybean seedlings without disease symptoms or insect spots were selected and sown in a polypropylene pot (upper diameter = 21 cm, lower diameter = 15 cm, and height = 19 cm). The pots were filled with peat-soil mixed with vermiculite at a volume ratio of 3:1 and pH 7.0 and maintained in a controlled environmental chamber with a light regime of 16 h/8 h (light/dark) and relative humidity of 50%–55% at 28 ± 2°C until the V1 stage. The seedlings were thinned to three per pot to obtain uniform seedlings and then treated with 50 ml of each CK (control), 75 mM NaCl (SS), 20% (W/V) PEG 6000 (D), and SS+D. After 5 days of treatment, the soybean root and shoot tissues were separated and collected in liquid nitrogen for 5 min, then stored at −80°C for RNA extraction and transcriptome analysis. The soybean seedlings in the same pot/petri dish were considered one experimental unit. All experiments were repeated three times.

### 2.3 RNA extraction, transcriptome analysis, and gene expression by qRT-PCR

Total RNA of soybean root and leaf samples were extracted using the Trizol reagent (Invitrogen, CA, USA), and their quality and purity were checked using the NanoDrop 2000 (Thermo Fisher Scientific, Wilmington, DE). The RNA Nano 6000 Assay Kit of the Agilent Bioanalyzer 2100 system (Agilent Technologies, CA, USA) was used to detect RNA integrity. The sequencing libraries were constructed by Biomarker Technologies Corporation (Beijing, China) on the Illumina HiSeq2500 as recommended by the manufacturer. After deleting the low-quality bases, the clean reads were mapped to the soybean genome (*Glycine max* Wm82.a2.v1). The differentially expressed genes (DEGs) with an adjusted *p*-value < 0.01 found by DESeq2 and FDR < 0.01 were assigned as differentially expressed.

The single-stranded cDNA of soybean seedling samples was synthesized using a 5 × HiScript SuperMix II according to the manufacturer’s (Vazyme, Nanjing, China) instructions. The *GmCKXs* primers (**Table S1**) were designed with Primer 5.0 (Primer, Canada). Soybean *TUBULIN A* (NM_001250372) and *ACTIN* (NM_001289231) were used as the internal control genes. The qRT-PCR reaction was conducted using SYBR qPCR Master Mix (Vazyme, Nanjing, China) and run using the Roche Cycler 480II system (Roche, Roche Diagnostics, Switzerland). Relative expression levels for each CKX gene were calculated using the operational formula 2^−ΔΔCt^ (Livak and Schmittgen, 2001). Three technical replicates and three biological replicates were performed for each reaction for each sample in this study.

### 2.4 Determination of CKX enzyme activity and zeatin content

The CKX enzyme activity of samples was detected using the ELISA kit (10894, Meibiao, Jiangsu, China) according to the instructions. The zeatin content was determined using high-performance liquid chromatography (HPLC-MS/MS) (AB SCIEX, ShimadzuLc-20AD, AB5500 Massachusetts, USA) at the Customs Quality Inspection Center (Dalian, Liaoning, China).

### 2.5 Statistical analysis

Results were analyzed using one-way analysis of variance (ANOVA) and the Duncan’s multiple range tests within the SPSS 19.0 (SPSS Inc., Chicago, IL, United States). Differences in values were considered statistically significant at *p* < 0.05.

## 3 Results

### 3.1 Identification and physicochemical property analysis of GmCKX genes in soybean

A total of 18 *GmCKX* genes were identified according to the result of an HMM profile, InterPro, and SMART analysis (**Table 1**). The results showed that the 18 GmCKX proteins contained amino acids (aa) ranging from 320 in *GmCKX06* to 552 aa in *GmCKX09*, with the lowest isoelectric point (IP) in *GmCKX11* (4.95) and the highest IP of 9.12 in *GmCKX07* and a low molecular weight (MW) of 35,800.26 Da in *GmCKX06* and a high MW of 62,281.19 Da in *GmCKX09*.

**Table 1:** Molecular characteristics of *GmCKX* genes in soybean.

Gene name	Gene_id	Chr	Location	Protein length (aa)	Isoelectric point	Molecular weight (Da)
*GmCKX01*	*Glyma.03G133300*	3	34850820/34853963	545	6.71	61,052.75
*GmCKX02*	*Glyma.04G028900*	4	2346613/2353642	424	6.23	48,279.08
*GmCKX03*	*Glyma.04G055600*	4	4492866/4496940	422	5.12	47,785.35
*GmCKX04*	*Glyma.06G028900*	6	2262638/2269432	424	5.87	48,345.8
*GmCKX05*	*Glyma.09G063500*	9	6102365/6107334	527	6.74	59,275.84
*GmCKX06*	*Glyma.09G063700*	9	6120899/6127022	320	5.87	35,800.26
*GmCKX07*	*Glyma.09G063900*	9	6163747/6168342	546	9.12	61,883.72
*GmCKX08*	*Glyma.09G225400*	9	45006788/45009855	534	6.37	60,063.71
*GmCKX09*	*Glyma.11G149100*	11	11564330/11568822	552	6.79	62,281.19
*GmCKX10*	*Glyma.12G011400*	12	831481/834416	538	6.24	60,336.71
*GmCKX11*	*Glyma.13G104600*	13	21926847/21931738	524	4.95	58,804.63
*GmCKX12*	*Glyma.13G104700*	13	21935494/21939535	535	7.35	60,986.77
*GmCKX13*	*Glyma.14G099000*	14	9505502/9511512	513	5.81	57,182.32
*GmCKX14*	*Glyma.15G170300*	15	15411545/15416020	543	8.73	61,546.2
*GmCKX15*	*Glyma.17G054500*	17	4143438/4147686	535	6.42	60,945.76
*GmCKX16*	*Glyma.17G054600*	17	4151290/4156276	522	5.34	58,758.95
*GmCKX17*	*Glyma.17G225700*	17	37956646/37963562	496	5.85	55,658.43
*GmCKX18*	*Glyma.19G135100*	19	39630174/39633290	545	6.12	60,841.21

**Figure 1:**
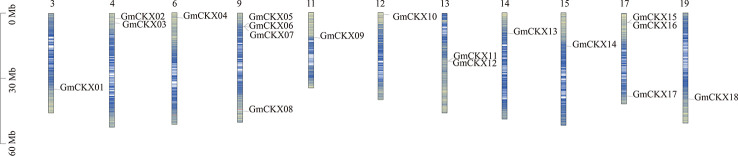
Localization of the *GmCKXs* on the soybean chromosomes. The chromosomal position of each *GmCKX* gene is shown on the corresponding chromosome from top to bottom according to the soybean genome. The blue line shows the gene density. The darker the color, the more dense the gene. The value on the *Y*-axis represents the position of the chromosome. The chromosome number is shown at the top of each bar.

As shown in **Figure 1**, 18 *GmCKX* genes were unevenly distributed on 11 chromosomes: one gene on chromosome 3 (5.56% of the total), two genes on chromosome 4 (11.11% of the total), one gene on chromosome 6 (5.56% of the total), four genes on chromosome 9 (22.22% of the total), one gene on chromosome 11 (5.56% of the total), one gene on chromosome 12 (5.56% of the total), two genes on chromosome 13 (11.11% of the total), one gene on chromosome 14 (5.56% of the total), one gene on chromosome 15 (5.56% of the total), three genes on chromosome 17 (16.66% of the total), and one gene on chromosome 19 (5.56% of the total). In addition, *GmCKXs* are mostly distributed at both ends of the chromosomes.

### 3.2 Phylogenetic analysis of GmCKX proteins

**Figure 2:**
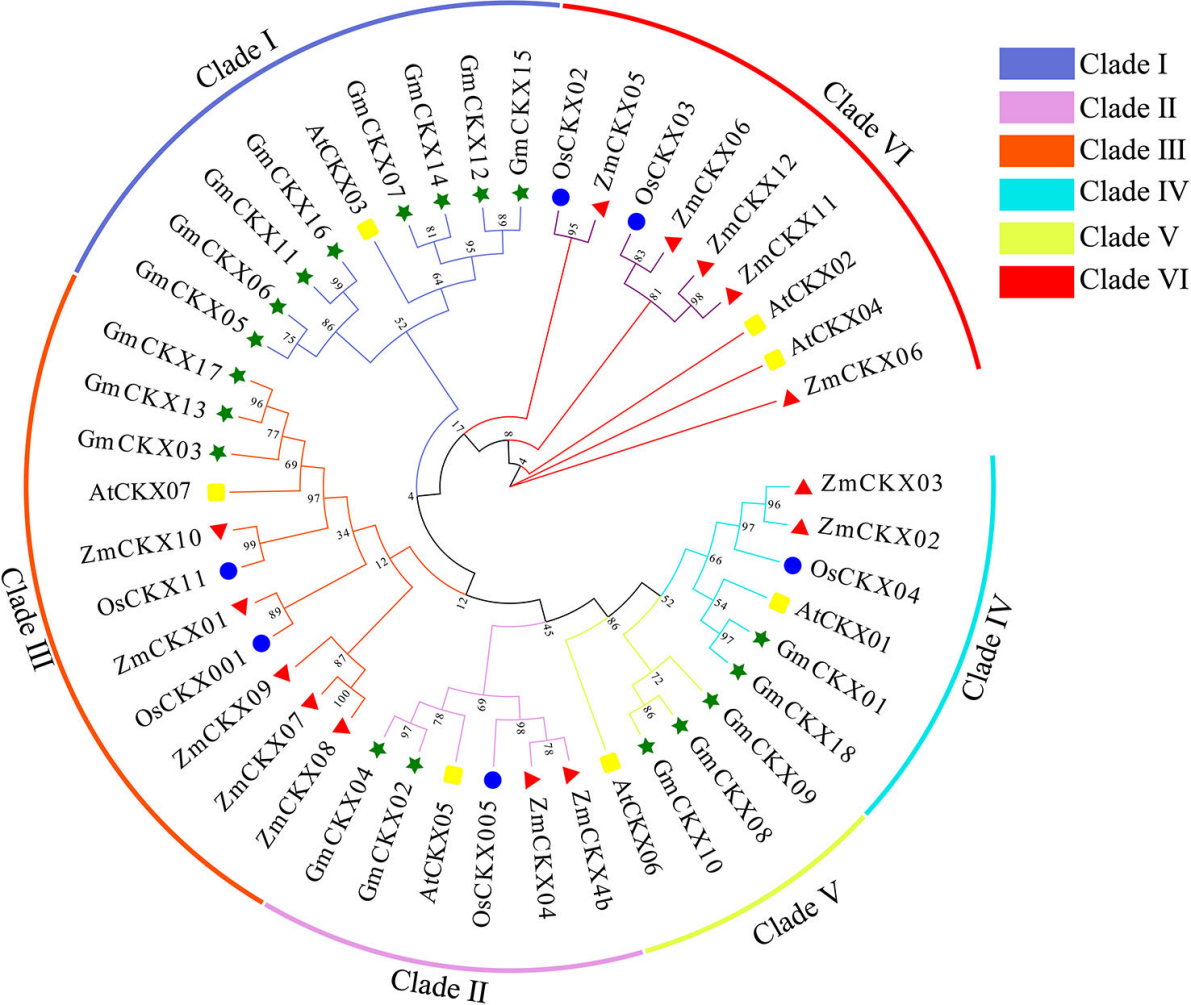
Phylogenetic analysis of CKX proteins in soybean, *Arabidopsis*, maize, and rice. The green, blue, red, and yellow circle represent soybean (*G. max* L.), *A. thaliana*, maize (*Z. mays* L.), and rice (*O. sativa* L.) respectively.

To understand the evolution and development of the *CKX* gene family members in different species, 7 AtCKX, 11 OsCKX, 13 ZmCKX, and the 18 GmCKX proteins were assessed in a phylogenetic tree (**Figure 2**). The different CKXs were divided into six major clades (I–VI), with those from soybean only distributed in five subfamilies. Among the GmCKX proteins, clade I contained eight proteins, namely, GmCKX5, GmCKX6, GmCKX7, GmCKX11, GmCKX12, GmCKX14, GmCKX15, and GmCKX16; clade II had GmCKX2 and GmCKX4; clade III contained GmCKX3, GmCKX13, and GmCKX17; clade IV had GmCKX1 and GmCKX18; and clade V contained GmCKX8, GmCKX9, and GmCKX10 (**Figures 2**, **S2**).

### 3.3 Conserved motifs and gene structure analysis

**Figure 3:**
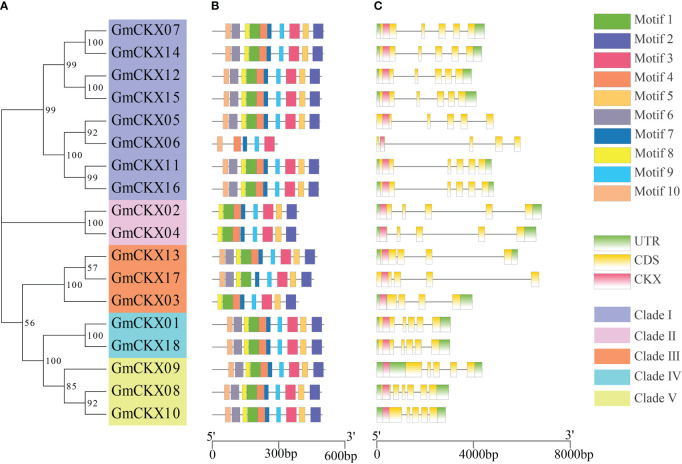
Gene structure and conserved motifs of the *GmCKXs*. **(A)** The phylogenetic classification of *GmCKXs*. **(B)** Conserved motif analysis of *GmCKXs*; different colored rectangles represented different motifs. **(C)** Gene structure analysis of *GmCKXs*. UTR regions (green rectangles), exons (yellow rectangles), CKX domains (pink rectangles), and introns (black lines).

The online software MEME was used to analyze the conservative motifs of GmCKXs. A total of 10 conserved motifs were obtained from the 18 GmCKXs, designated as Motifs 1 to 10 (**Figures 3A, B**). The *GmCKX* members in the same subfamily had similar motif characteristics but differed among *GmCKXs* in other subfamilies. Most *GmCKX* members contained 10 motifs each, with *GmCKX6* found in clade I containing five motifs, *GmCKX2* and *GmCKX4* (clade II), and GmCKX3 (clade III) containing eight motifs each. We analyzed the exon–intron structures of the *GmCKX* members on the GSDS website (**Figure 3C**) and found similar exon–intron structures for *GmCKXs* in the same subfamily, with differences among *GmCKXs* in different subfamilies. The number of exons in the 18 *GmCKX* genes ranged from four to seven, with the majority containing six exons each. The least number of exons was found in *GmCKX3*, while the highest number was found in *GmCKX8*.

### 3.4 Analysis of promoter *cis*-regulatory elements

**Figure 4:**
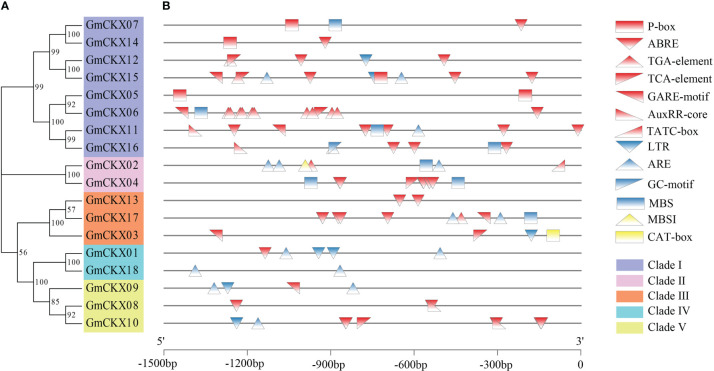
Promoter *cis*-regulatory element analysis of the *GmCKXs*. **(A)** The phylogenetic classification of *GmCKXs*. **(B)** *Cis*-element analysis of the promoter regions of *GmCKXs* genes. P-box: gibberellin-responsive element; ABRE: abscisic acid elements; TGA-element: auxin responsive element; TCA-element: salicylic acid elements; GARE-motif: gibberellin responsive element; AuxRR-core: auxin response promoter element; TATC-box: gibberellin responsive element; LTR: low-temperature responsiveness; ARE: anaerobic responsiveness; GC-motif: enhancer-like element involved in anoxic specific inducibility; MBS: drought stress inducibility element; MBSI: flavonoid biosynthesis regulation; CAT-box: meristem expression element.

To further study the regulatory mechanism of the *GmCKX* family in response to abiotic stress, the upstream 1.5-kb sequences of each of the 18 *GmCKXs* were extracted and used to analyze the *cis*-regulatory elements (**Figure 4**; **Table S2**). We identified 13 *cis*-regulatory elements and divided them into three groups: hormone-, resistance-, and physiological metabolism-related elements. The hormone-related elements consisted of the P-box, ABRE, TGA-element, TCA-element, GARE-motif, AuxRR-core, and TATC-box. The resistance-related elements included LTR, ARE, GC-motif, and MBS, while the physiological metabolism-related elements had only the MBSI and CAT-box.

### 3.5 Collinearity analysis

**Figure 5:**
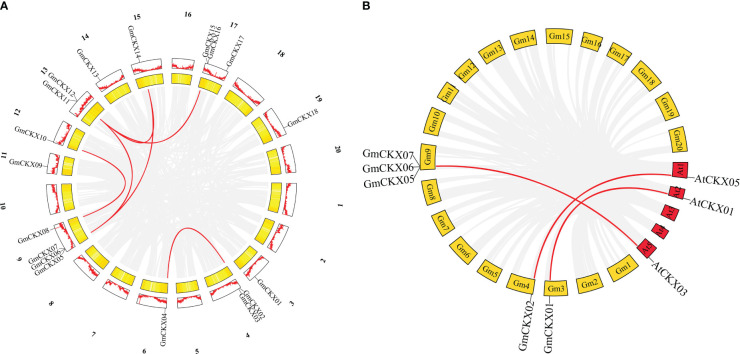
Collinearity analysis of the *GmCKXs*. **(A)** Duplicated gene pairs in soybean genome. Red lines indicate the duplication of *GmCKXs* gene pairs. **(B)** Collinearity analysis of *GmCKX* genes with *A. thaliana*. Red lines connect fragments of repeated gene pairs between soybean and *A*. *thaliana*.

The origins of duplicates for *CKX* genes were detected by MCScanX and used to analyze the distribution and arrangement of its homologs within or between species. We identified six *GmCKX* duplicate gene pairs in the soybean genome, all characterized as segmental duplication events (**Figure 5A**; **Table S3**). Subsequently, we performed nonsynonymous and synonymous substitution ratio (Ka and Ks) analyses of duplicated genes to examine the driving forces of the soybean CKX gene family. The results showed that all six *GmCKX* gene pairs underwent purification selection with the Ka/Ks < 1. The collinearity analysis of *GmCKXs* with *Arabidopsis* was further used to explore the evolutionary mechanisms of the soybean CKX gene family. The results identified five orthologous gene pairs between soybean and *Arabidopsis* as collinear pairs, including *GmCKX1/AtCKX1*, *GmCKX2/AtCKX5*, *GmCKX5/AtCKX3*, *GmCKX6/AtCKX3*, and *GmCKX6/AtCKX3* (**Figure 5B**).

### 3.6 Expression profile analysis of *GmCKX* genes in soybean tissues

**Figure 6:**
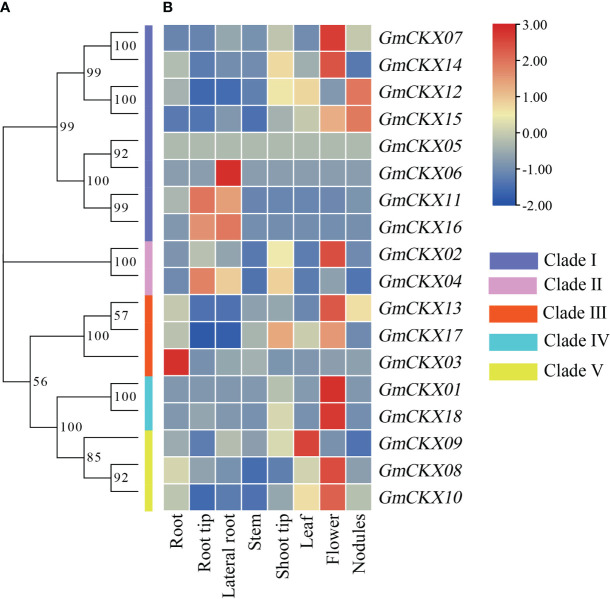
Heatmaps of the expression profiling of 18 *GmCKX* genes. **(A)** The phylogenetic classification of *GmCKXs*. **(B)** Expression profiling of *GmCKXs* in different tissues based on the Phytozome database. The color scale represents expression levels from high (red) to low (blue).

**Figure 7:**
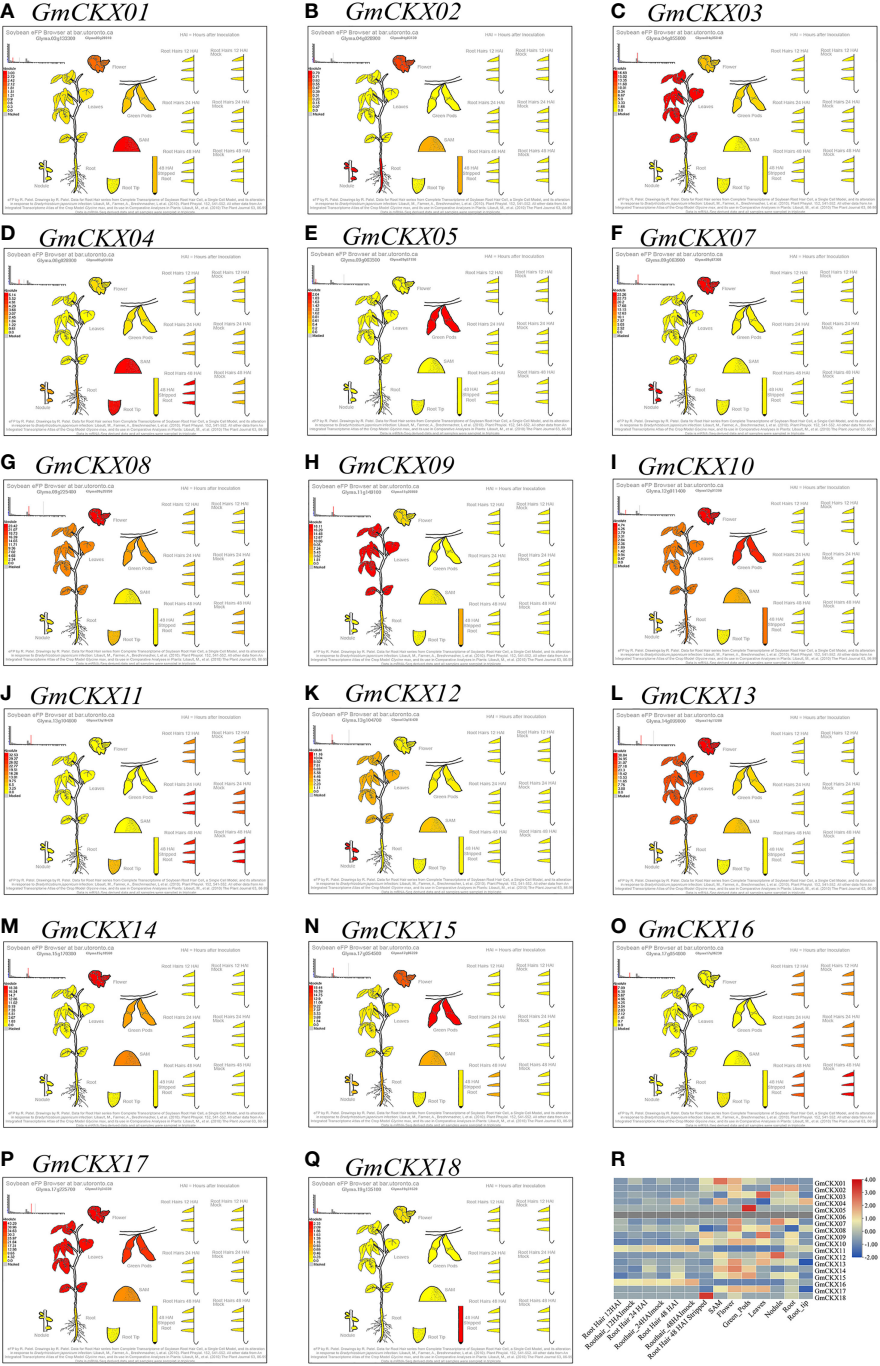
Expression profiling of *GmCKXs* in different tissues based on the Soybean eFP Browser database. **(A–Q)** Diagram showing the different soybean tissues. Red represents high expression and yellow represents low expression. **(R)** The heatmap were drawn by TBtools. Red represents high expression and blue represents low expression.

To explore the spatiotemporal expression patterns of soybean *GmCKX* genes, we compared the transcript abundances of all the 18 *GmCKX* genes using two publicly available RNA-Seq data from the Phytozome and Soybean ePF Browser database, respectively (**Figures 6**, **7**). The Phytozome dataset contained root, root tip, lateral root, stem, shoot tip, leaf, flower, and nodules. In contrast, the Soybean ePF Browser dataset consisted of the root hair, shoot apical meristem (SAM), flower, green pods, leaf, nodule, root and root tip, and also the treatment and control root hair tissue after *Bradyrhizobium japonicum* infection at three different time points (Libault et al., 2010a; Libault et al., 2010b). Most *GmCKX* genes were preferentially expressed in more than one tissue, with *GmCKX7* and *GmCKX8* highly expressed and *GmCKX5* and *GmCKX6* genes lowly expressed or undetected in both two datasets. The data also showed that over 40% of the highly expressed *GmCKX* genes occurred in the floral organs of soybean.

### 3.7 Expression analysis of *GmCKX* genes in soybean seeds during the germination stage

**Figure 8:**
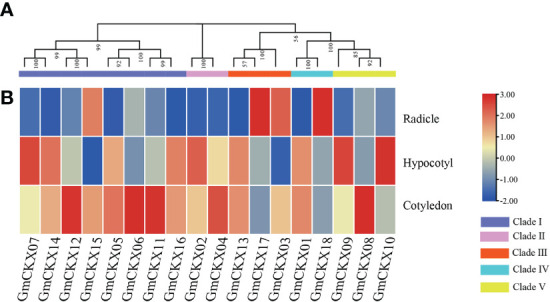
Expression pattern of soybean *GmCKX* genes in different tissues by qRT-PCR analysis during the germination stage. **(A)** The phylogenetic classification of *GmCKXs*. **(B)** Expression profiling of *GmCKXs* in radicle, hypocotyl, and cotyledon tissues. The color scale represents expression levels from high (red) to low (blue).

Because seed germination is an important growth stage in the plant life cycle, we used qRT-PCR to investigate the expression of *GmCKXs* in the soybean seed’s radicle, hypocotyl, and cotyledon during this stage (**Figure 8**). Some genes displayed tissue-specific expression. For example, *GmCKX3*, *GmCKX17*, and *GmCKX18* were found in the radicle; *GmCKX7, GmCKX9*, and *GmCKX10* were found in hypocotyl; and *GmCKX6*, *GmCKX8*, *GmCKX11*, and *GmCKX12* were found in the cotyledon.

### 3.8 Expression patterns of *GmCKXs* under abiotic stress

**Figure 9:**
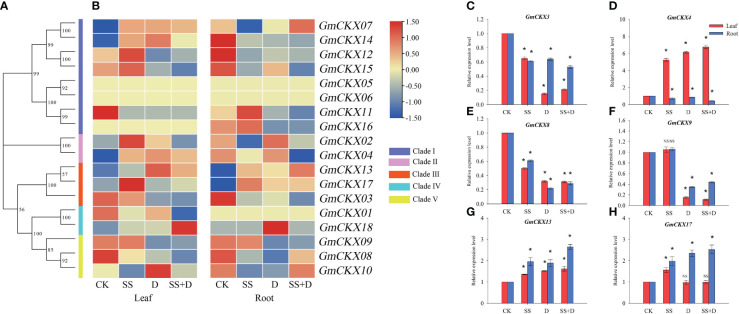
Expression profiles of soybean *GmCKXs* under different abiotic stresses at the seedling stage. **(A)** The phylogenetic classification of *GmCKXs*. **(B)** Expression profiling of *GmCKXs* in leaf and root under different abiotic stresses. CK, control condition; SS, salt stress; D, drought stress; SS+D, salt combined with drought stress. The color scale represents expression levels from high (red) to low (blue). **(C–H)** qRT-PCR analysis of six selected *GmCKX* genes in leaf and root under different abiotic stresses. Values are the means ± standard deviations of three replicates. * indicates significant difference between CK and treatment condition (*p* < 0.05). NS indicates no significant differences between CK and treatment condition.

To explore the roles of specific *GmCKX* genes in response to different abiotic stresses, transcriptome expression patterns of all soybean *GmCKX* genes were analyzed in the leaves and roots of soybean seedlings under salt (SS), drought (D), and salt combined with drought stress (SS+D) (**Figures 9A, B**). The assembled gene dataset was deposited at the National Center for Biotechnology Information with the accession number PRJNA930177. Our data showed different expression profiles of *GmCKX* genes in different stress treatments and tissues. For example, compared to the control, three stress treatments significantly upregulated the expression level of *GmCKX13* in leaf and root but downregulated the expression level of *GmCKX3* and *GmCKX8*. The *GmCKX14* gene in soybean leaves was highly upregulated under the three stress treatments, while its expression level in the roots was significantly downregulated. The expression levels of *GmCKX9* in leaf and root were downregulated considerably under D and SS+D treatment, but SS treatment had no significant effects on its expression level. In the leaf, the *GmCKX15* gene was highly expressed under SS treatment but downregulated under D and SS+D treatment. At the same time, all three stress treatments significantly downregulated the expression of *GmCKX15* in the roots. The accuracy of transcriptome data was verified by qRT-PCR of six randomly selected *GmCKX* genes (**Figures 9C–H**).

**Figure 10:**
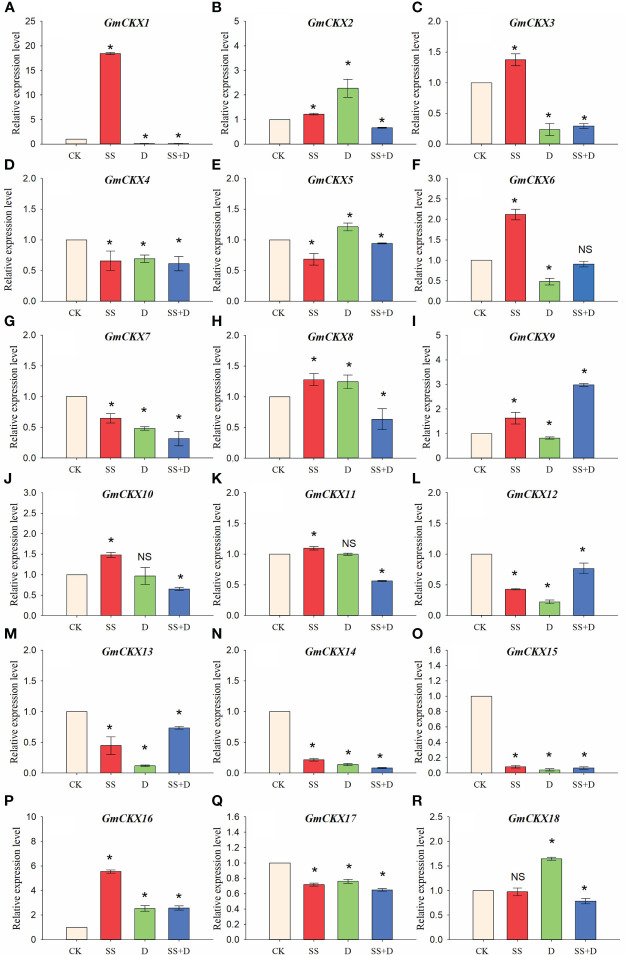
Relative expression levels of soybean *GmCKX* genes under SS, D, and SS+D treatments at the germination stage **(A–R)**. Values are the means ± standard deviations of three replicates. * indicates significant difference between CK and treatment condition (*p* < 0.05). NS indicates no significant differences between CK and treatment condition.

To further investigate whether *GmCKXs* participate in response to the abiotic stresses during the germination stage, soybean seed radicles treated with SS, D, and SS+D were collected for qRT-PCR (**Figure 10**). We found that the expression of most *GmCKX* genes differed under different stress treatments. For example, compared with the control, the expression of *GmCKX1* and *GmCKX3* was significantly upregulated under SS treatment but significantly downregulated under D and SS+D treatments. The *GmCKX2* and *GmCKX8* were upregulated considerably under SS and D treatments but were significantly downregulated under SS+D treatment. All three stress treatments also significantly downregulated genes such as *GmCKX4*, *GmCKX7*, *GmCKX12*, *GmCKX13*, *GmCKX14*, *GmCKX15*, and *GmCKX17*, but significantly upregulated *GmCKX16*.

### 3.9 Hormone-induced patterns of expression of the *GmCKX* genes

**Figure 11:**
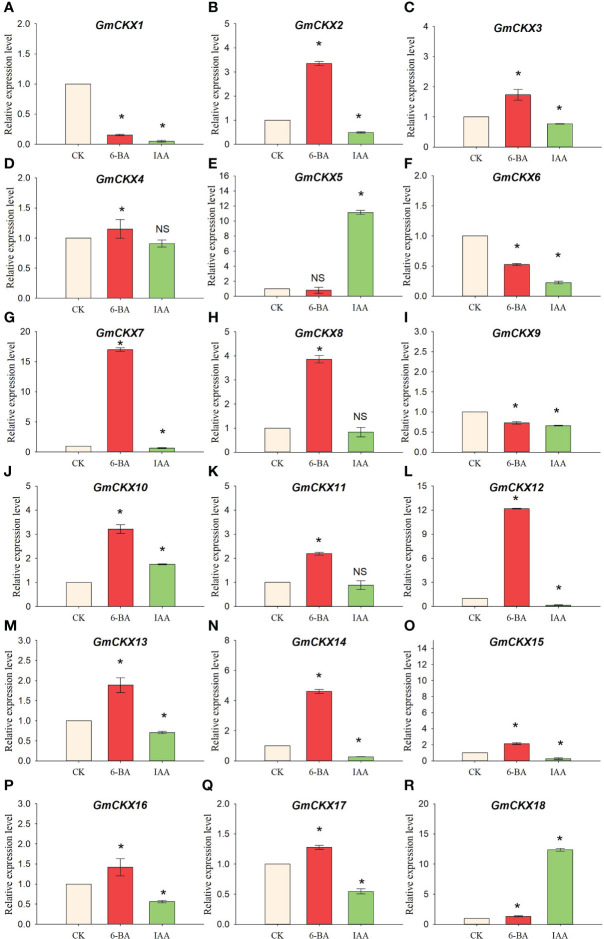
Relative expression levels of soybean *GmCKX* genes under IAA and 6-BA treatments **(A–R)**. Values are the means ± standard deviations of three replicates. * indicates significant difference between CK and treatment condition (*p* < 0.05). NS indicates no significant differences between CK and treatment condition.

We analyzed the relative expression level of *GmCKXs* in radicles treated with 6-BA and IAA using qRT-PCR to explore the hormone-induced patterns of expression of the *GmCKX* genes (**Figure 11**) and found differential expression of the *GmCKXs* under different hormone treatments. Compared to the control, the 6-BA and IAA significantly upregulated *GmCKX10* and *GmCKX18* genes but highly repressed the *GmCKX1, GmCKX6*, and *GmCKX9* genes. The *GmCKX2*, *GmCKX3*, *GmCKX7*, *GmCKX12*, *GmCKX13*, *GmCKX14*, *GmCKX15*, *GmCKX16*, and *GmCKX17* genes were upregulated considerably under 6-BA treatment but downregulated under IAA treatment. The *GmCKX4, GmCKX8*, and *GmCKX11* genes were significantly upregulated under 6-BA treatment but remained unaffected under IAA treatment. The IAA hormone also affected the expression of *the GmCKX5* gene by significantly upregulating it.

### 3.10 Abiotic stress and hormone-induced changes of CKX enzyme activity and zeatin content

**Figure 12:**
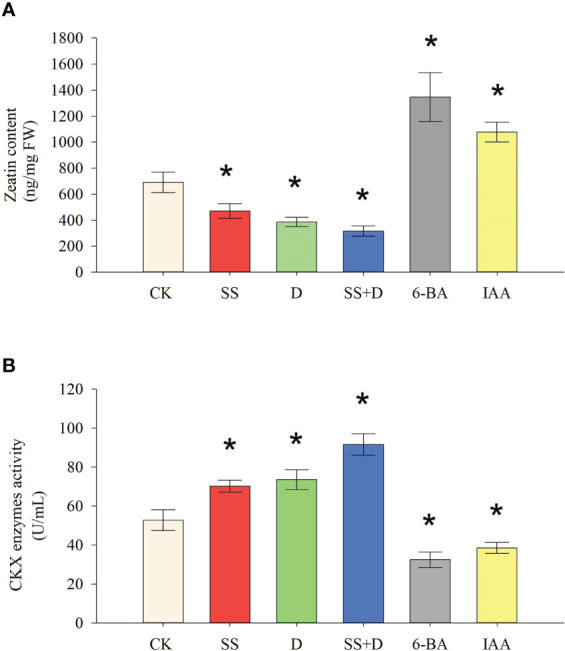
Zeatin content and CKX enzyme activity under different abiotic stresses and hormone treatments. **(A)** Zeatin content under SS, D, SS+D, 6-BA, and IAA treatments. **(B)** CKX enzyme activity under SS, D, SS+D, 6-BA, and IAA treatments. Values are the means ± standard deviations of three replicates. * indicates significant difference between CK and treatment condition (*p* < 0.05). NS indicates no significant differences between CK and treatment condition.

We determined the zeatin content and CKX enzyme activity in soybean radicles under SS, D, SS+D, 6-BA, and IAA treatments to analyze the relationship between CKX enzyme activity and CTK content under abiotic stress (**Figure 12**). Compared with the control, SS, D, and SS+D stress treatments significantly decreased zeatin content in soybean radicles by 32.02%, 44.2%, and 54.31%, respectively. In comparison, 6-BA and IAA treatments significantly increased the zeatin content in soybean radicles by 274.79% and 199.81%, respectively. Compared to the control, SS, D, and SS+D significantly increased CKX enzyme activity in soybean radicles by 32.9%, 39.35%, and 73.46%, respectively, while 6-BA and IAA significantly decreased CKX enzyme activity in soybean radicles by 38.33% and 26.9%, respectively.

## 4 Discussion

CTKs play an important role in numerous plant physiology processes, such as fatty acid biosynthesis in seed (Thien Nguyen et al., 2016), the development of plant floral organs and pod setting (Nonokawa et al., 2012), leaf senescence (Merewitz et al., 2010), and seed yield (Jameson and Song, 2016; Chen et al., 2020b). The hormone also helps the plants to respond to a variety of abiotic stresses, including drought (Hai et al., 2020), heat (Prerostova et al., 2020), and salt (Yu et al., 2022). Several members of the *CKX* gene family, which regulate the endogenous CKs, have been identified in various crops, including 11 members in rice (Mameaux et al., 2012), 13 in maize (Zalabák et al., 2014), 36 in cabbage (Zhu et al., 2022), 23 in oilseed rape (Liu et al., 2018), 5 in potato (Suttle et al., 2014), and 12 members in apple (Tan et al., 2018). In this study, we identified 18 *CKX* family members with complete domains in the entire genome of soybean, named *GmCKX1*-*GmCKX18*, according to their position on the chromosome. The variation in the number of *CKX* genes in different species is possibly due to genome evolution and replication, which causes the production of homologous genes and an increase in their numbers (Kaltenegger et al., 2018; Wang et al., 2021). We further divided the soybean CKXs into five subgroups (I–V) based on a phylogenetic tree containing *Arabidopsis* and soybean CKX protein sequences. Detailed molecular characterization of the *GmCKX* genes showed that CKX protein members had different physicochemical properties, such as protein length, pI, and MW, indicating a high diversity of these gene family members. These insights will help investigate the function of *GmCKXs*.

The exon–intron structure provides important information for exploring the evolutionary relationship of genes (Ahmad et al., 2019). In general, the number of exons plays an important role in gene evolution (Xu et al., 2012), and the number of introns determines the rate of gene expression (Jeffares et al., 2008; Shaul, 2017). Genes with similar exon–intron structures have similar gene functions. Therefore, gene function can be predicted by analyzing its structure (Li et al., 2019b). In the current study, most *GmCKX* genes in the same subfamilies had similar numbers of exons and introns, suggesting that they might have similar functions. However, we also found some *GmCKX* genes with different exon and intron numbers within the same subfamily, a phenomenon that has also been reported in oilseed rape (Liu et al., 2018) and cabbage (Zhu et al., 2022), which might be due to the functional diversity of genes throughout evolution.

The chromosomal localization analysis showed that the *GmCKX* genes were distributed non-homogeneously on chromosomes, showing a cluster distribution, which may be attributed to the non-uniform replication event of soybean chromosome fragments. Gene duplication is an important mechanism that promotes the expansion and diversification of gene families. Synteny analyses revealed that segmental duplication contributed to the expansion and diversification of the soybean *GmCKX* gene family. Similar results have been observed in maize (Gu et al., 2010) and Chinese cabbage (Liu et al., 2013). The study also showed that all six *GmCKX* gene pairs had Ka/Ks < 1, indicating that *GmCKXs* underwent purification selection under environmental stress, which is consistent with the results of previous studies (Yu et al., 2021; Zhu et al., 2022). Genome comparison is considered a relatively fast and effective method to study the potential characteristics and functions of genes (Lyons and Freeling, 2008). Therefore, the possible role of *CKX* homologous genes in the soybean can be inferred by analyzing the information on *CKX* genes in the model plant, such as *Arabidopsis*. This is supported by the location of five orthologous gene pairs in syntenic genomic regions between soybean and *Arabidopsis* genomes. For example, the *atCKX1* gene is expressed in root tissues and participates in lateral root formation (Chang et al., 2015). Ectopic expression of the *AtCKX1* gene in tobacco enhanced drought and heat stress tolerance (Macková et al., 2013). In *Arabidopsis*, *AtCKX3* and *AtCKX5* genes were expressed in reproductive meristems. The *ckx3 ckx5* double mutant could delay the differentiation of reproductive meristem cells and exhibit more and larger flower and silique numbers (Bartrina et al., 2011). Based on the reported function of the CKX gene (*AtCKX1*, *AtCKX3*, and *AtCKX5*) in *Arabidopsis*, we could predict the possible role of the soybean *GmCKX* genes. However, their functional roles need to be further confirmed in future reverse genetics studies.

Germination is the initial stage of soybean growth, which is also the most sensitive to environmental stress. The expression levels of genes in tissues and organs are closely related to their functions. We used two sets of public databases and qRT-PCR data from germination soybeans to detect the expression levels of the 18 *GmCKX* genes in different soybean tissues. The expression of the *GmCKX* genes differed among the soybean tissues, indicating that the *GmCKX* genes had different biological functions and were involved in soybean growth regulation and various tissue development processes. The expression patterns of individual *GmCKXs* in soybean were shown to be tissue and development specific. For example, *GmCKX7* and *GmCKX8* were highly expressed in all organs, while *GmCKX5* was mainly expressed in hypocotyl and cotyledon, and *GmCKX6* was highly expressed in cotyledon. These results are consistent with studies of *CKX* genes in other species (Song et al., 2012; Chen et al., 2020b; Yu et al., 2021).

CTK is a physiological hormone that widely exists in plants. As a key enzyme, which degrades endogenous CTK, CKX plays an important role in maintaining intracellular CTK homeostasis and for adaption to environmental stress (Vyroubalová et al., 2009; Le et al., 2012). *Cis*-acting elements play an important role in signal transduction and regulation of gene transcription initiation. Analysis of *cis*-acting elements in the promoters of *GmCKXs* demonstrated that the *GmCKXs* play a role in response to the hormone, plant growth, and biotic and abiotic stress responses. Similar findings were found in the *CKX* gene families in oilseed rape, maize, and *Arabidopsis*. For example, the overexpression of *MsCKX* genes in *Arabidopsis* exhibited stronger salt tolerance (Li et al., 2019a), while *ZmCKX1* was strongly induced by CTKs, abscisic acid, and abiotic stress in maize (Brugière et al., 2003). In oilseed rape, the expression level of *BnCKX7-1* was downregulated by the exogenous supply of 6-BA (Liu et al., 2018). In the current study, soybean *GmCKX* genes showed various roles in response to salt, drought, salt combined with drought stresses, 6-BA, and IAA. Our results demonstrate that each *GmCKX* gene is expressed differently in response to salt and drought stress and the exogenous supply of 6-BA and IAA hormones. The analysis of the *GmCKX* gene family at seedling and germination stages of soybean under salt, drought, and salt combined with drought stress showed that *GmCKX14* was downregulated both in root at the seedling stage and in hypocotyl at the germination stage under the three abiotic stress treatments. The results of the evolutionary analysis showed that *GmCKX14* and *AtCKX3* were homologous genes. The overexpression of the *AtCKX3* gene increased the growth rate of primary roots and reduced the number of flowers in transgenic *Arabidopsis* (Werner et al., 2003). Transgenic tomatoes with overexpressed *AtCKX3* gene maintained the plants in a higher water state by reducing transpiration under drought treatment, thus enhancing their drought resistance (Farber et al., 2016). Our results suggested that soybean *GmCKX14* might be an important negative regulatory gene in abiotic stress such as salt and drought. The analysis of zeatin content and CKX enzyme activity in radicle under salt, drought, and salt combined with drought stress confirmed that abiotic stress enhanced CKX enzyme activity but reduced zeatin content.

In bread wheat, exogenous hormone treatment significantly induced *TuCKXs* gene expression within 3 h (Shoaib et al., 2019). Our results show that an exogenous supply of 6-BA and IAA could dramatically reduce the CKX enzyme activity of soybean radicle and enhance zeatin content. However, the soybean *GmCKXs* showed different expression patterns in response to 6-BA and IAA. After the exogenous supply of 6-BA and IAA, the *GmCKX1*, *GmCKX6*, and *GmCKX9* genes were all repressed, while the *GmCKX10* and *GmCKX18* genes were upregulated. Combined with physiological analysis results, the *GmCKX1*, *GmCKX6*, and *GmCKX9* genes could be used as positive regulatory factors, and *GmCKX10* and *GmCKX18* could be used as negative regulatory factors to participate in CK metabolism in response to exogenous 6-BA and IAA. However, this regulatory effect still requires further confirmation of its functional role in future reverse genetics studies. Our results also provide a reference for studying the function of the *CKX* gene under abiotic stress and hormonal regulation.

## 5 Conclusion

In this study, the 18 *GmCKX* genes were identified from the soybean genome, and their evolutionary relationship, chromosomal location, gene structure, motifs, *cis*-regulatory elements, collinearity, and gene expression patterns were analyzed by bioinformatics tools, RNA-seq, and qRT-PCR methods. The *GmCKXs* members were divided into five clades according to the phylogenetic tree. Synteny analyses revealed that the expansion of the *GmCKXs* gene family is mainly due to fragment replication. The analysis of Phytozome and Soybean ePF Browser databases and qRT-PCR showed that *GmCKX* genes had tissue-specific expression patterns. In addition, *GmCKXs* genes were differentially regulated in response to salt, drought, salt combined with drought stress, 6-BA, and IAA treatments. Expression of *GmCKX14* was downregulated both in root at the seedling stage and in radicle at the germination stage under salt, drought, and salt combined with drought stress treatments. Under 6-BA and IAA treatments, the expressions of *GmCKX1*, *GmCKX6*, and *GmCKX9* decreased, while the expressions of *GmCKX10* and *GmCKX18* increased. Finally, physiological analysis results showed that *GmCKX* genes could respond to abiotic stress and regulate the activity of CKX enzymes and the zeatin content.

## Data availability statement

The datasets presented in this study can be found in online repositories. The names of the repository/repositories and accession number(s) can be found below: https://www.ncbi.nlm.nih.gov/, PRJNA930177.

## Author contributions

YD, ZZ, YG, JD, and QZ participated in the experimental design. YD, ZZ, WL, WW, XY, YZ, and MY performed material sampling, gene expression experiments, and physiology experiments. YD, ZZ, YG, and JD contributed to the data collection and data analysis. YD wrote the manuscript. ZZ, JD, and QZ revised the manuscript. All authors contributed to the article and approved the submitted version.
