# Supplementary material for: Genome-wide identification of the soybean cytokinin oxidase/dehydrogenase gene family and its diverse roles in response to multiple abiotic stress
Source: Front Plant Sci. 2023 Apr 17;14:1163219. doi: 10.3389/fpls.2023.1163219 (PMC10149856; doi:10.3389/fpls.2023.1163219)
Supplement: Supplementary file 1 [file DataSheet_1.docx]

Supplementary Material

Article Title Genome-wide identification of soybean cytokinin oxidase/dehydrogenase (CKX) gene family and its diverse roles in response to multiple abiotic stress

Yanli Du^1,4 #^, Zhaoning Zhang^1, #^, Yanhua Gu ^1^, Weijia Li ^1^, Weiyu Wang ^1^, Xiankai Yuan ^1^, Yuxian Zhang ^2,4^, Ming Yuan^5^, Jidao Du ^1,3,4*^, and Qiang Zhao ^2,3*^

*** Correspondence:** Jidao Du [djdbynd@163.com](mailto:djdbynd@163.com)); Qiang Zhao

[zqiang0416@hotmail.com](mailto:zqiang0416@hotmail.com)

Supplementary Figures and Tables

Figure S1. Chemical structure of CTK.

Figure S2. Phylogenetic tree of GmCKX proteins in soybean.

Table S1: Specific primers for qRT-PCR.

Table S2: Analysis and function prediction of *cis*-regulatory elements of *GmCKX* genes.

Table S3: Identification of substitution rates for homologues *GmCKX* genes.

## Supplementary Figures


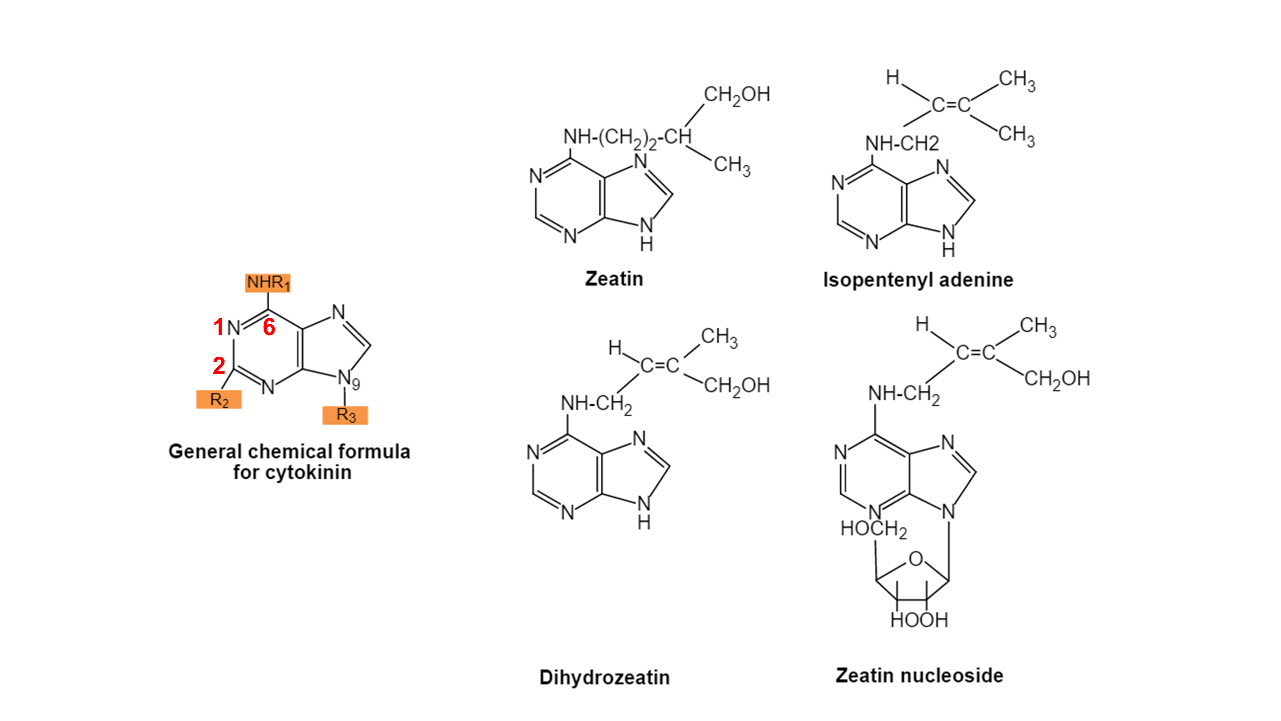


Figure S1. Chemical structure of CTK.


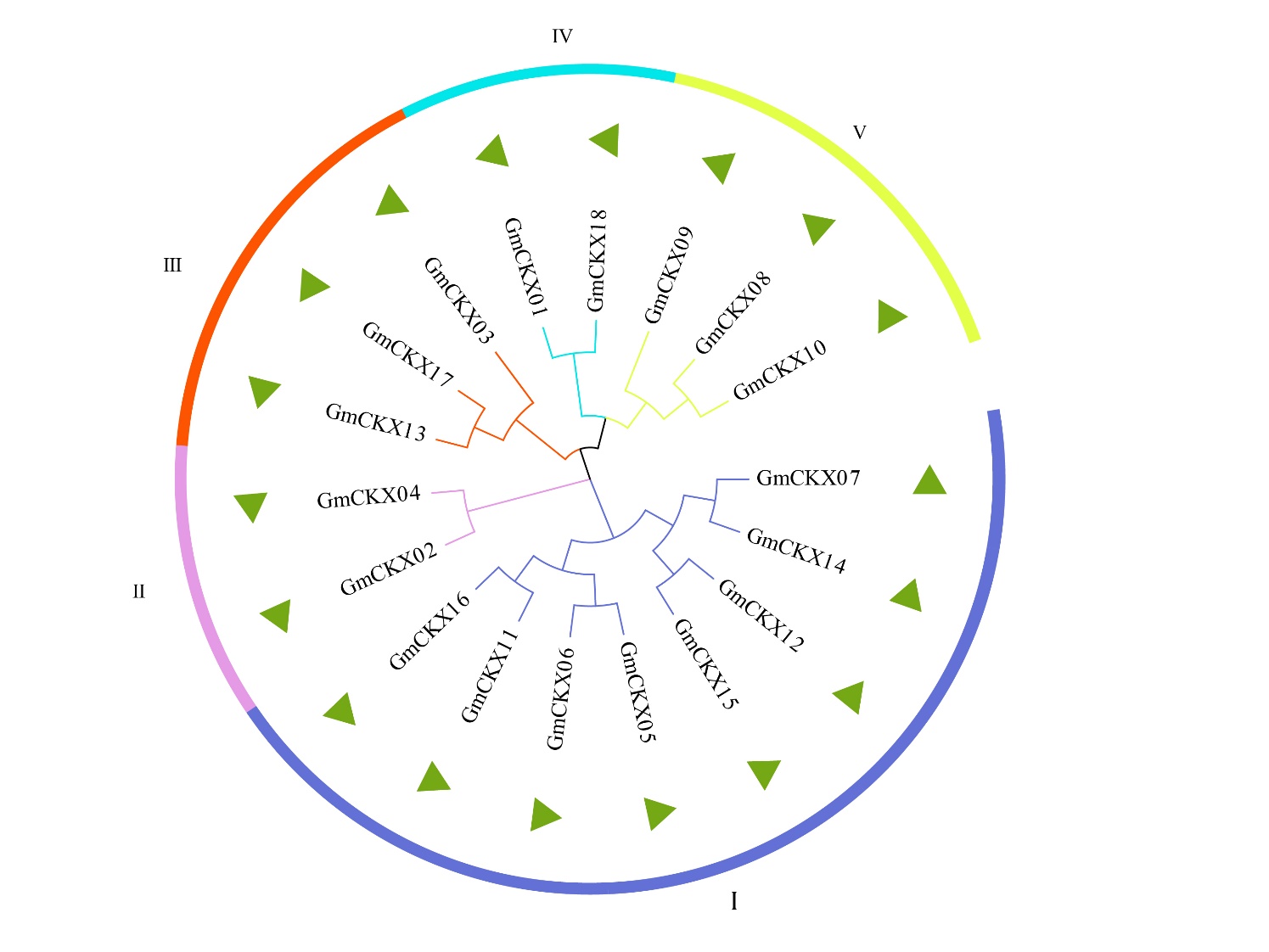


Figure S2. Phylogenetic tree of GmCKX proteins in soybean.

## Supplementary Tables

Table S1: Specific primers for qRT-PCR

| Primer | Sequence (5 'to 3') | Primer | Sequence (5 'to 3') |
| --- | --- | --- | --- |
| GmCKX01_F | TGAAGGTGCACGAAGTTTCACTTG | GmCKX11_F | TCTGTGCGAATGCTGGTATTGGG |
| GmCKX01_R | AATTATTGAGAGCAAGGCCAGAGG | GmCKX11_R | ACTGCTTCTCCCATTCTTCCTGTG |
| GmCKX02_F | AGTGGACCCAGTTCAATGCTAGG | GmCKX12_F | GCTCCAAGGACTAGCCTATATCCC |
| GmCKX02_R | TGCTGGCCTGTGGCTAAGATTC | GmCKX12_R | CCACAGTCCTTGTGACTGAAGC |
| GmCKX03_F | ACGGGAGGATTGGATCAAGCAC | GmCKX13_F | TCGTAACCAAGACCATCCTTCCG |
| GmCKX03_R | TTGGCCTTTCGCTCCGCAAATC | GmCKX13_R | TTAGACCCTCGACGAATCGTAGCC |
| GmCKX04_F | TGGTGAAGGCAGGGTTCAAGTC | GmCKX14_F | ACGTTGCTGGGATCAAGGTTAAG |
| GmCKX04_R | GCTTGCCCGTTTATCGAATGTCC | GmCKX14_R | GCCCAATCTTCTTGTGTGCGATAG |
| GmCKX05_F | TGCTTAGGTCAGAAGGACTTTGGG | GmCKX15_F | AAACTCCATGATGACCCTGAAACC |
| GmCKX05_R | TCTTGGAACCCAGAGGTTCAGC | GmCKX15_R | GCGTTAAATGGCCATAGTCCCTTG |
| GmCKX06_F | TGTTGCCGAGCTAGTTCTTAGGC | GmCKX16_F | CAGGGCAAGAATAGCTCTTGGG |
| GmCKX06_R | CCAGATGTTCAACCAAGGATGAGG | GmCKX16_R | GAGAAATCGTTGTACAGCAAACGG |
| GmCKX07_F | CAATGGATGAAGGCCCTAACGC | GmCKX17_F | AGGCCAGTGAGATGGATAAGGG |
| GmCKX07_R | AGCTTGCTGAAGATGGTGTCATCG | GmCKX17_R | ACTCTGCGTCTCGAGTGAAGTC |
| GmCKX08_F | ACTACCTGCATCTCACAGTTGGTG | GmCKX18_F | ACCATCGTTGGTTACTCCAGAGG |
| GmCKX08_R | GGGACCATGCCTAAATGTCTGACC | GmCKX18_R | TTGGGAGTGCTGAGGATAGGAGTG |
| GmCKX09_F | ATACACCATTTCGCAGAGGTTGTC | TUBULIN A_F | AGGTCGGAAACTCCTGCTGG |
| GmCKX09_R | AGGGCCGTTGCTTGTTTCTTTAAC | TUBULIN A_R | AAGGTGTTGAAGGCGTCGTG |
| GmCKX10_F | AACTGGAACTGACGGCCTAGAG | ACTIN_F | CGGTGGTTCTATCTTGGCATC |
| GmCKX10_R | TCTTGCTGTGTGTTGTAGTGAGG | ACTIN_R | GTCTTTCGCTTCAATAACCCTA |

Table S2: Analysis and function prediction of *cis*-regulatory elements of *GmCKX* genes

| Element | Core sequence | Classification | Function |
| --- | --- | --- | --- |
| P-box | CCTTTTG | Hormone related elements | gibberellin-responsive element |
| ABRE | ACGTG | Hormone related elements | cis-acting element involved in the abscisic acid responsiveness |
| TGA-element | AACGAC | Hormone related elements | auxin-responsive element |
| TCA-element | CCATCTTTTT | Hormone related elements | cis-acting element involved in salicylic acid responsiveness |
| GARE-motif | TCTGTTG | Hormone related elements | gibberellin-responsive element |
| AucRR-core | GGTCCAT | Hormone related elements | cis-acting regulatory element involved in auxin responsiveness |
| TATC-box | TATCCCA | Hormone related elements | cis-acting element involved in gibberellin-responsiveness |
| LTR | CCGAAA | Resistance-related elements | cis-acting element involved in low-temperature responsiveness |
| ARE | AAACCA | Resistance-related elements | cis-acting regulatory element essential for the anaerobic induction |
| GC-motif | CCCCCG | Resistance-related elements | enhancer-like element involved in anoxic specific inducibility |
| MBS | CAACTG | Resistance-related elements | MYB binding site involved in drought-inducibility |
| MBSI | aaaAaaC(G/C)GTTA | Resistance-related elements | MYB binding site involved in flavonoid biosynthetic genes regulation |
| CAT-box | GCCACT | Development-related element | cis-acting regulatory element related to meristem expression |

Table S3: Identification of substitution rates for homologues *GmCKX* genes

| Gene pairs | Non-synonymous substitution rates (Ka) | Synonymous substitution rates (Ks) | Ka/Ks |
| --- | --- | --- | --- |
| GmCKX04/ GmCKX 02 | 0.01693 | 0.106003 | 0.159717 |
| GmCKX 12/ GmCKX 07 | 0.137202 | 0.898054 | 0.152777 |
| GmCKX 14/ GmCKX 07 | 0.024484 | 0.109258 | 0.22409 |
| GmCKX 10/ GmCKX 08 | 0.023345 | 0.143643 | 0.162521 |
| GmCKX 15/ GmCKX 11 | 0.319037 | 1.395714 | 0.228583 |
| GmCKX 14/ GmCKX 12 | 0.134548 | 0.909953 | 0.147862 |
